# Enhanced Unidirectional Cell Migration Induced by Asymmetrical Micropatterns with Nanostructures

**DOI:** 10.3390/jfb16090323

**Published:** 2025-09-01

**Authors:** Kaixin Chen, Yuanhao Xu, Stella W. Pang

**Affiliations:** 1 Department of Electrical Engineering, City University of Hong Kong, Hong Kong, China; kaixichen7-c@my.cityu.edu.hk (K.C.); yuanhaoxu2@cityu.edu.hk (Y.X.); 2 Centre for Biosystems, Neuroscience, and Nanotechnology, City University of Hong Kong, Hong Kong, China

**Keywords:** nanofabrication, unidirectional cell migration, nanostructures, osteoblast cells, fibronectin, asymmetrical patterns

## Abstract

Directed cell migration is crucial for numerous biological processes, including tissue regeneration and cancer metastasis. However, conventional symmetrical micropatterns typically result in bidirectional cell migration guidance instead of unidirectional guidance. In this study, polydimethylsiloxane (PDMS)-based platforms with asymmetrical arrowhead micropatterns, nanopillars, and selective fibronectin coating were developed to enhance unidirectional cell migration. The platforms were fabricated using nanoimprint lithography and PDMS replication techniques, allowing for precise control over surface topography and biochemical modification. The MC3T3 osteoblastic cells cultured on these platforms demonstrated significantly enhanced directional migration, characterized by increased displacement, and directional alignment with micropattern orientation compared to symmetrical patterns. Quantitative analyses revealed that asymmetrical arrowheads combined with nanopillars induced more focal adhesions and F-actin polarization at cell front regions, supporting the observed unidirectional cell migration enhancement. These results confirm that integrating micropattern asymmetry, nanoscale features, and biochemical functionalization synergistically promotes unidirectional cell migration. The developed platforms offer valuable insights and practical strategies for designing advanced biomaterials capable of precise spatial cell guidance that can be applied to the designs of organ-on-a-chip systems.

## 1. Introduction

Cell migration is a fundamental biological process in which cells actively move from one location to another in response to environmental stimuli [1,2,3]. As one of the most extensively studied phenomena in cell biology, the mechanisms and regulation of directed cell migration have been widely investigated [4,5,6,7]. To better understand and to precisely control directed cell migration, researchers have developed various guiding strategies, such as chemical gradients, electrical fields, substrate stiffness gradients, and topographical cues, to achieve effective contact guidance [8,9,10,11,12,13,14,15]. Symmetrical micropatterns, including linear grooves or ridges, are commonly used to induce cell alignment, thereby enhancing migration velocity and directional migration [9,16,17,18,19,20,21,22,23]. However, due to their inherent geometric symmetry, such patterns typically result in bidirectional cell migration, characterized by frequent directional reversals and limited persistence in one specific direction.

Unidirectional cell migration requires stable cell polarization, a condition challenging to achieve using symmetric topographical features that lack the spatial biases needed to establish and maintain cell polarity [9,24]. Conversely, asymmetrical micropatterns provide directional cues capable of promoting cytoskeletal polarization and generating the biased traction forces essential for unidirectional cell migration [21,25]. Furthermore, nanoscale topographies can significantly influence cellular behaviors by enhancing focal adhesion (FA) formation and modulating cytoskeletal dynamics, thereby improving cell migration efficiency [15,26,27,28,29,30]. Focal adhesions, as mechanosensitive structures, mediate mechanical signals between the actin cytoskeleton and the extracellular matrix (ECM) through integrin receptors, facilitating the traction force transmission necessary for cell motility [31,32,33,34,35,36]. F-actin polymerizes at the cell front region to drive protrusions and retracts at the back region to facilitate forward motion as the major cytoskeletal component [37,38,39,40]. The distribution and quantitative analysis for FAs and F-actin are thus essential for directed cell migration. Polydimethylsiloxane (PDMS) is frequently used to form platforms for cell migration due to its tunable surface properties and biocompatibility [41,42,43]. Additionally, fibronectin (FN), a crucial ECM protein that engages integrin receptors and links to intracellular actin networks, can be selectively coated onto PDMS to enhance cellular adhesion and guidance, further refining control over cell behaviors [44,45,46,47].

Microchannel-based platforms are widely used to study cell migration under confined conditions and have been particularly useful for evaluating cell invasiveness or drug responses [48,49,50]. However, microchannels inherently impose strong physical constraints on cells, often restricting their ability to form broad lamellipodia or to establish spontaneous cell polarity. As a result, there is limitation to reveal how cells intrinsically sense and respond to environmental guidance cues in these platforms. By contrast, this study aims to investigate how cells sense and respond to asymmetrical surface topographies and biochemical cues. By removing physical constraints, these platforms enable cells to develop spontaneous polarity, directional bias, and unidirectional migration. The asymmetrical arrowhead patterns provide unidirectional topographical cues, while the nanopillars mimic ECM-like features that modulate adhesion formation and cytoskeletal organization. The current platforms isolate the effects of surface geometry from physical confinement and allow for a direct examination of how cells interact with topographical surfaces.

In this study, the effects of symmetrical and asymmetrical micropatterns, nanoscale features, and selective biochemical modification on cell migration direction were systematically investigated. PDMS platforms featuring symmetrical gratings and asymmetrical arrowhead patterns, with or without integrated nanopillars or selective FN coating, were fabricated and compared. Through quantitative analyses of FA localization and F-actin distribution, it was demonstrated that asymmetrical arrowhead micropatterns integrated with nanopillars and selective FN coatings could enhance cell polarization and significantly promote unidirectional cell migration. These findings reveal the cooperative roles of micropattern asymmetry, nanoscale topography, and biochemical signaling, offering valuable insights into designing advanced biomaterial interfaces for targeted and controlled cell migration.

## 2. Materials and Methods

### 2.1. Nanostructures Patterning on Guiding Micropatterns by Nanoimprint Lithography

**Figure 1:**
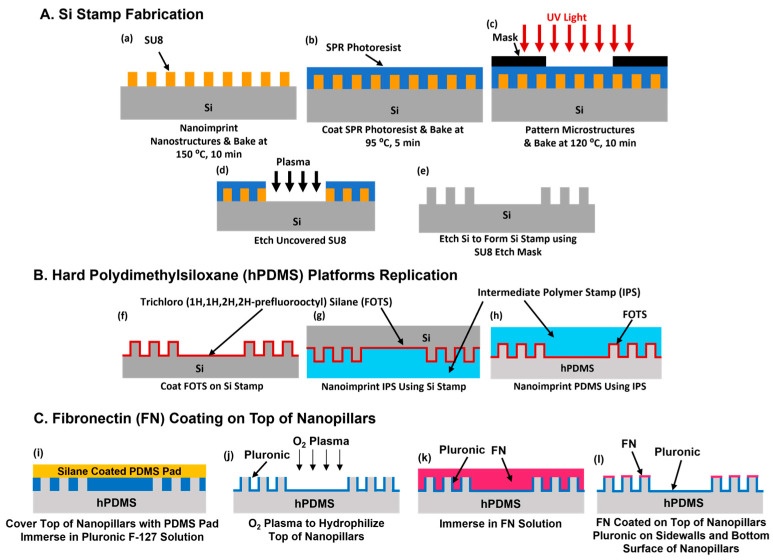
Schematics of fabrication technology for micropatterns with nanostructures and fibronectin (FN) coating on top of nanopillars. (**a**–**e**) Silicon (Si) stamp fabrication including micropatterns with nanostructures by nanoimprinting SU8 nanopillars, followed by optical lithography to form micropatterns with SU8 as an etch mask. (**f**–**h**) Polydimethylsiloxane (PDMS) platforms replicated by nanoimprinting intermediate polymer stamp (IPS) using an Si stamp and nanoimprinting PDMS using an IPS stamp. (**i**–**l**) FN selectively coated on top of nanopillars with Pluronic coated on sidewalls and bottom surfaces of nanopillars.

The fabrication of micropatterns with nanostructures was mainly divided into two parts. The first part was the Si stamp fabrication, as illustrated in Figure 1a–e. An intermediate polymer stamp (IPS) containing a 500 nm deep nanohole was coated with trichloro(1H,1H,2H,2H-perfluorooctyl) silane (FOTS, J&K Scientific, San Jose, CA, USA) at 80 °C for 25 minutes (min). This coating improved the stamp separation in the subsequent nanoimprint lithography (NIL). SU8 photoresist was then nanoimprinted to form nanopillars on the Si substrate using the IPS stamp. The process was carried out at 90 °C under a pressure of 30 bar, followed by ultraviolet (UV) exposure for 60 s and baking at 150 °C for 10 min to solidify the nanostructures. Next, a layer of SPR6112B photoresist was spin coated over the SU8 nanopillars at 3000 rotations per min (rpm) for 60 s and baked at 95 °C for 5 min. The substrate was then exposed to UV light for 15 s and baked again at 120 °C for 10 min. This process defined the grating and arrowhead micropatterns on top of the nanopillars. Reactive ion etching (RIE) was used to selectively remove the exposed SU8 nanopillars. The SU8 etching was performed under a pressure of 10 mTorr with 120 W RF power and a gas flow of 20/2 sccm SF_6_/O_2_. The remaining SU8 nanopillars served as an etch mask for RIE to form nanopillars in the Si stamp. The dry etching was conducted at 28 mTorr with 600 W RF coil power, 14.8 W RF platen power, and 138/11 sccm SF_6_/O_2_ gas flow for 3 min to a depth of 500 nm.

The replication of micropatterned platforms in PDMS was shown in Figure 1f–h. A silane coated Si stamp was used to fabricate an IPS replica by NIL. The IPS was then treated with FOTS to facilitate the subsequent demolding of the PDMS structures. Hard polydimethylsiloxane (hPDMS) consisting of a silicone elastomer base/curing agent (Gelest, Inc., Morrisville, PA, USA) in a 1:1 weight ratio was spin coated on a glass substrate. The patterned IPS was pressed into the hPDMS layer and baked at 80 °C for 2 h to cure the material. After curing, the IPS was peeled away, transferring the micropatterned nanostructures into the hPDMS layer. To enhance cell attachment, the surface of the hPDMS replica was treated with an oxygen plasma with 20 mTorr at 5 sccm O_2_ and 80 W RF power for 90 s. This treatment increased the surface hydrophilicity, improving compatibility with the cell culture.

The method for selective FN coating on nanostructures is shown in Figure 1i–l. The hPDMS platform was immersed in 0.2% Pluronic F-127 solution for 1 h. A silane coated PDMS pad consisting of a silicone elastomer base/curing agent (SYLGARD™ 184, Dow, Stade, Germany) in a 10:1 weight ratio was placed on top to cover the nanopillars, preventing them from being coated by the Pluronic. After removing both the Pluronic solution and the PDMS pad, the platform was placed inside a Plasma Asher with a Faraday cage. It was treated with a nitrogen and oxygen mixture at 300 mTorr with 27 W RF power for 20 s. This step increased the surface energy at the exposed nanopillar tops without disturbing the Pluronic coating [51]. An FN solution at 50 μg/mL was applied for 15 min. The FN concentration of 50 μg/mL exceeds the reported monolayer saturation level of 10 μg/mL on cell culture substrates [52]. The higher concentration ensures complete surface coverage and allows for a consistent evaluation of topography-induced migration responses. FN selectively adhered to the top of the nanopillars, while the Pluronic coating prevented FN adsorption on the sidewalls and bottom surfaces. This resulted in a platform with FN coated only on the top of the nanopillars, while the sidewalls and base remained uncoated. No additional plasma treatment was applied to the FN coating on top of the nanopillars of the arrowheads before cell seeding.

In addition, fetal bovine serum containing FN was added as a supplement into DMEM as the cell culture medium. For platforms without an FN coating, any nonspecific protein adsorption from the medium would occur for all the topographical platforms after the plasma treatment, as identical medium composition and volume were used in each experiment. In these cases, cells migrated based on the topographical guidance cues. For the platforms with the FN coating on top of the nanopillars of the arrowheads, selectively coated FN attracted more cells to migrate on the coated regions, as the surrounding hPDMS regions were coated with Pluronic which prevented nonspecific protein adsorption and cell adhesion. Consequently, cell migration on the FN coated nanopillars of the arrowheads was influenced by both the topographical cues and the selective FN coating.

### 2.2. Cell Culture and Cell Seeding

MC3T3-E1 osteoblastic cells CRL-2594 (American type culture collection, Manassas, VA, USA) were cultured in high glucose Dulbecco’s Modified Eagle Medium. This medium was supplemented with 10% fetal bovine serum (FBS, Gibco, Waltham, MA, USA), and 2 mM alanyl-l-glutamine (GlutaMAX™, Gibco, Waltham, MA, USA). Cells were maintained at 37 °C in a humidified atmosphere with 5% CO_2_. To ensure optimal cell motility and viability, the culture medium was replaced every 2 days. Cells were passaged before the confluency reached 80% to maintain healthy growth conditions. Before cell seeding, hPDMS platforms were sterilized by washing twice with 75% ethanol and 1× phosphate saline buffer (PBS, Life Technologies, Carlsbad, CA, USA). The MC3T3 cells were then seeded onto the platforms at a density of 1 × 10^4^ cells/mL. The cultures were incubated for 6 h to allow for cell attachment. Once the cells had fully adhered to the surface, the medium was replaced with a CO_2_-independent medium. The platform was washed twice with 1× PBS before the medium was changed. The timelapse imaging was performed over a 16-h period under these conditions.

### 2.3. Time-Lapse Imaging and Data Processing

Cells on the hPDMS platforms were maintained at 37 °C and imaged every 5 min for a total duration of 16 h using a Nikon microscope (Eclipse Ni-E, Nikon, Tokyo, Japan) equipped with a 20× magnification objective lens and a numerical aperture of 0.75. The migration of the MC3T3 cells was tracked using the Manual Tracking plugin in ImageJ (version 1.53v, National Institutes of Health, Bethesda, MD, USA). To ensure an unbiased analysis of cell migration behavior, only cells that had no interaction with other cells throughout the observation period were included in the analysis. Cells that divided, died, or interacted with neighboring cells were excluded. Data were collected from at least three independent sets of experiments to ensure reproducibility and robustness. The aspect ratio of each cell was calculated as the ratio of the major axis to the minor axis, providing a quantitative measure of cell elongation. Statistical comparisons between groups were performed using one-way ANOVA and Tukey’s post hoc test [53]. The mean and ± standard error of the mean were displayed for each dataset.

### 2.4. Immunofluorescence Staining and Confocal Microscopy

After timelapse imaging, the MC3T3 cells were washed twice with 1× PBS for 5 min each. Cells were then fixed by using 4% paraformaldehyde (PFA, Sigma-Aldrich, St. Louis, MO, USA) for 15 min. Following fixation, the samples were washed twice with 1× PBS to remove residual fixative. Cells were permeabilized with 0.1% Triton X-100 (Thermo Fisher Scientific, Waltham, MA, USA) for 15 min. After another two washes with 1× PBS, nonspecific binding was blocked with 1% bovine serum albumin (Thermo Fisher Scientific, Waltham, MA, USA) for 30 min. For FA, cells were then incubated with mouse anti-vinculin primary antibody (Merck, Rahway, NJ, USA)/1× PBS in a 1:200 volume ratio for 12 h. After two 10-min washes with 1× PBS, samples were incubated with FITC-conjugated goat anti-mouse IgG secondary antibody (Merck, Rahway, NJ, USA)/1× PBS in a 1:500 volume ratio for 2 h. To visualize the cytoskeleton, F-actin was stained with TRITC-conjugated Phalloidin (Merck, Rahway, NJ, USA)/1× PBS in a 1:200 volume ratio for 2 h. Cell nuclei were stained with 4′,6-diamidino-2-phenylindole (DAPI, Merck, Rahway, NJ, USA)/1× PBS in a 1:500 volume ratio for 30 min. Each staining step was followed by two 1× PBS washes to remove excess reagents. Immunofluorescence imaging was performed using a confocal microscope (Stellaris 8, Leica Microsystems, Wetzlar, Germany) equipped with a 40× oil objective. Fluorescence signals of the nucleus, vinculin, and F-actin were captured using 405, 488, and 532 nm excitation lasers, respectively, and shown in blue, green, and red colors. Reflected bright field images were obtained using a 650 nm laser.

### 2.5. Focal Adhesion and F-Actin Analysis in MC3T3 Cells

To quantify the size, number, and spatial distribution of FAs per cell, fluorescence images of vinculin staining were processed using ImageJ. Each cell was divided into front and back regions by bisecting the cell body along its long axis. The long axis was determined by the cell morphology using the Fit Ellipse function in ImageJ. The front region typically had broader lamellipodia or larger cell area [54,55]. Vinculin images were converted to binary format, and individual FAs were identified and analyzed based on their location, count, and area. F-actin organization was assessed using phalloidin-stained images. They were also converted to binary format, and the F-actin area was measured within each cell. The F-actin fraction was calculated as the ratio of the F-actin area to the total cell spreading area, providing a measure of actin enrichment relative to the size of cell body.

## 3. Results

### 3.1. Guiding Patterns with Nanostructures and Fibronectin Coating

Cells respond sensitively to both mechanical and biochemical cues in their environment, which can strongly influence their motility and migration directionality [4,20,56]. In this study, nanostructures and the biological modification of surface topography were used to investigate the impact of different guiding patterns on MC3T3 cell migration direction. To ensure an effective comparison across different topographical platforms, the periodicity of the grating and arrowhead patterns were designed to have a consistent 5 μm width and 5 μm spacing grating arrangement. These designs allow for a direct comparison of cell behavior on symmetrical versus asymmetrical topographies. The regular grating pattern provides continuous and linear guidance, whereas the arrowhead pattern introduces asymmetrical pattern densities which are higher near the arrowhead tips. They enable a controllable investigation into how topographical asymmetry modulates cellular polarization and promotes unidirectional migration. To further examine the synergistic effects of nanoscale topography, nanopillars were selectively integrated with the guiding patterns. Importantly, neither the PDMS substrate nor the nanopillar structures restricts cell movement. Therefore, to compare the continuous cell guidance effect achieved by these symmetrical and asymmetrical topographies, an FN coating was applied on top of the nanopillars of the arrowhead patterns. This biochemical modification serves to study the effect of the FN coating on cell adhesion and migration on guiding topography, which could reinforce unidirectional movement.

**Figure 2:**
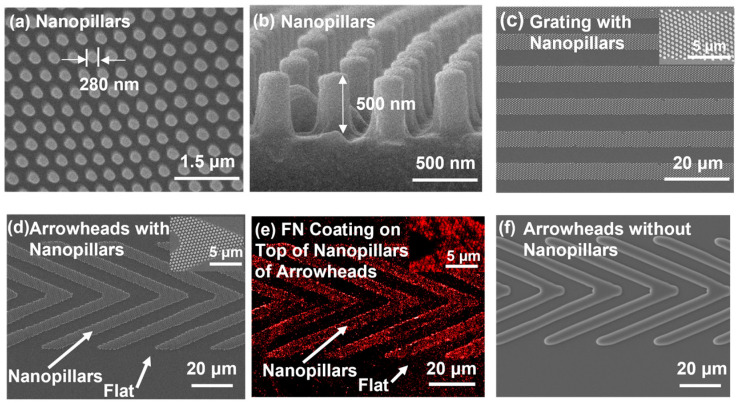
Micrographs of (**a**,**b**) nanopillars with 280 nm diameter, 535 nm pitch, and 500 nm height, (**c**) 5 μm wide and 5 μm spacing grating with nanopillars, (**d**) 45°, 50 μm wide, 50 μm spacing between rows, 60 μm long arm length, and 5 μm spacing between arms for arrowheads with nanopillars, (**e**) FN coating on top of nanopillars of arrowheads (red: FN with rhodamine fluorescence), and (**f**) arrowheads without nanopillars.

To promote cell migration activity, nanopillars in dimensions of 280 nm diameter, 535 nm pitch, and 500 nm height were fabricated, as shown in Figure 2a,b. The hPDMS platforms were prepared with two types of micropatterns. The first type of hPDMS patterned platforms, including 5 μm wide parallel ridges and 5 μm wide spacing gratings, were integrated with nanopillars, as shown in Figure 2c. The second type of platforms featured asymmetrical arrowhead patterns with a tip angle of 45°, 50 μm wide, 50 μm spacing between rows, 60 μm long arm length, and 5 μm spacing between arms, as shown in Figure 2d–f. To assess the influence of nanoscale and biochemical cues, the arrowhead patterns with and without nanopillar integration were compared. Additionally, platforms with the selective FN coating on top of the nanopillars of the arrowheads were generated to investigate its role in guiding cell migration. The MC3T3 cells on these guiding patterned platforms were also compared with those cultured on flat and nanopillar surfaces.

### 3.2. Effects of Nanopillars, Guiding Patterns with Nanopillars, and Fibronectin Coating on Cell Migration Trajectories

Previous studies have demonstrated that guiding patterns can influence cell migration directionality [17,21]. However, asymmetrical arrowhead patterns with and without nanostructure integration and FN modification have not been investigated. In this study, cells on the flat and the nanopillar surface were observed. MC3T3 cells were selected as the cell line due to their responsiveness to surface topography, substrate stiffness, and mechanical cues [57,58]. Their migratory behaviors and adhesion dynamics have been studied, making them a desirable model for investigating topography-guided cell motility. Future work will include other epithelial and mesenchymal cells to strengthen the universality and translational potential of topography-guided cell migration strategies.

**Figure 3:**
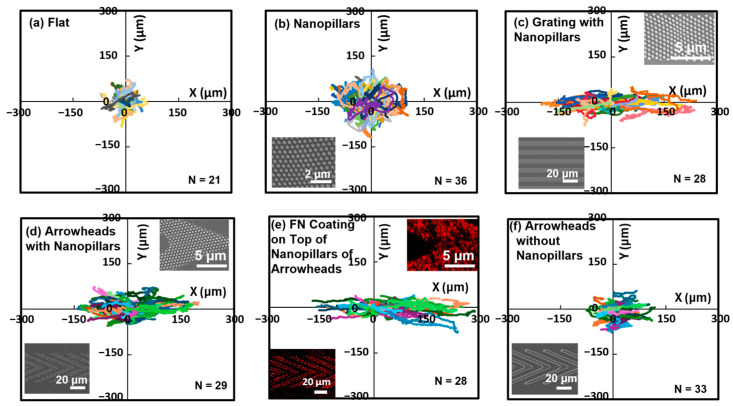
Migration trajectories of MC3T3 cells on (**a**) flat, (**b**) nanopillar, (**c**) grating with nanopillar, (**d**) arrowheads with nanopillar, (**e**) FN coating on top of nanopillars of arrowhead (red: FN with rhodamine fluorescence), and (**f**) arrowheads without nanopillar surfaces. Each color represents the trajectory of a single cell.

As shown in Figure 3a,b, cells on flat and hPDMS surfaces with nanopillars exhibited random migration directions. This behavior is attributed to the isotropic nature of these surfaces. Although nanopillars increased the cell migration distance by providing additional contact area for cell adhesion, they did not induce directional movement. On nanopillars in a grating arrangement, cell trajectories aligned along the grating orientation, as shown in Figure 3c. This guided migration resulted from cell elongation along the grating ridges, consistent with contact guidance mechanisms induced by symmetrical topography [17].

There was a slight decrease in the cell migration distance, and the cell migration trajectories were shifted moderately towards the arrowhead tip direction on the arrowheads integrated with nanopillars compared to the grating with nanopillars, as shown in Figure 3d. A larger increase in the trajectory length and path towards the tips of the arrowhead was observed for the arrowheads with nanopillars coated with FN, as shown in Figure 3e. Compared to the arrowheads with nanopillars, the FN coating further enhanced the trajectory length and unidirectional migration in one single direction, suggesting a synergistic effect induced by the nanostructures and biochemical cues. This significant unidirectional guidance effect was not observed when only nanopillars were applied. This suggests that biochemical modification on the nanopillar surface can further guide migration by attracting cells along the tips of the arrowheads. These findings confirm that unidirectional cell migration can be guided by asymmetrical topographies. Nanopillars integrated with micropatterns enhance migration distance, while an FN coating on top of the nanostructures improves directionality through additional biochemical cues. Arrowhead patterns without nanopillars also promoted directional migration, as shown in Figure 3f, showing more aligned trajectories compared to the random movement on flat or nanopillar surfaces. The asymmetrical arrowhead micropattern alone can serve as directional cues for cell migration [20]. However, the trajectory length on this platform was less than that on the arrowheads with nanopillars. It is reasonable that nanopillar modification provided more surface contact area for cell adhesion, enabling faster and longer distances for cells to migrate on nanostructures in a microscale arrangement. Notably, even without the FN coating, the arrowheads with and without nanopillars demonstrated cell migration guidance in a single direction along the arrowhead tips while the gratings provided guidance for cells to migrate in both directions. These results suggest that the asymmetrical topography of the arrowheads have the unique capability to maintain unidirectional migration guidance. The tips of the arrowheads introduce a denser surface contact area that could preferentially orient lamellipodia extension toward the tips, thereby enhancing the cell movement in a single direction. This indicates that the asymmetrical geometry alone can provide unidirectional cell migration guidance.

### 3.3. Cell Migration Motility Influenced by Nanopillars and Fibronectin Modification

**Figure 4:**
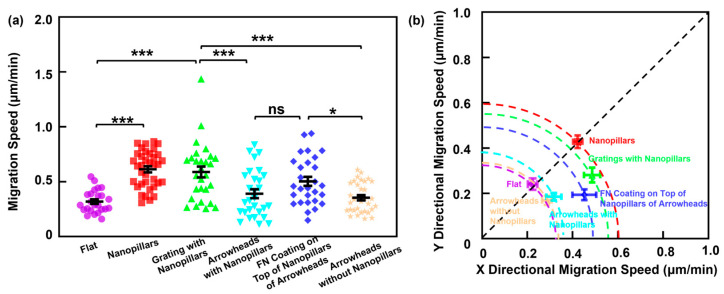
(**a**) MC3T3 cell migration speed on flat, nanopillar, grating with nanopillar, arrowheads with nanopillar, FN coating on nanopillars of arrowhead, and arrowheads without nanopillar surfaces. Each point represents a single cell. One-way ANOVA and Tukey’s post hoc test with * *p* < 0.05 and *** *p* < 0.001. (**b**) MC3T3 cell migration speed in x and y directions over 16 h, ns—not significant. Different colors correspond to groups of data on various platforms.

Figure 4a shows cell the migration speed across the platforms with different surface conditions. Cells on nanopillar platforms exhibited the highest speed of 0.61 μm/min. This is attributed to the increased surface contact area provided by the dense nanostructures, which facilitates cell movement. Cells on nanopillars in the grating arrangement demonstrated a slightly lower speed of 0.56 μm/min. This performance reflects the combined effects of topographical guidance from the grating and the enhanced cell adhesion from the nanopillars. The migration speeds on the arrowhead patterns with and without nanopillars were 0.37 and 0.35 μm/min, respectively. The minor difference suggests that the sparse arrowhead arrangement imposes limited spatial confinement, regardless of nanopillar integration. However, a significant increase in migration speed was observed for the arrowhead patterns with the FN coated nanopillars, reaching 0.50 μm/min. This result indicates that the FN coating on top of nanopillars enhances cell adhesion and promotes faster movement by confining cells along the intended migration path with the increased surface contact area by the nanopillars interaction. For all platforms with patterned topographies, the cell migration speed was higher than the speed on the flat surface.

To investigate the directional guidance effect of the patterned platform, cell migration speed along the x and y directions are shown in Figure 4b. Directionality was assessed by calculating the alignment angle between the net migration vector and the axis of the pattern [59]. A smaller angle represents greater alignment with the pattern orientation. An angle of 45° corresponds to equal speeds along both axes, indicating isotropic, non-directional migration. Cells on the flat and nanopillars surfaces aligned near the 45° reference line. This supports previous observations of random migration behavior on these surfaces, despite differences in overall speed. Among these isotropic platforms, cells on flat surfaces showed the lowest migration speeds, while those on nanopillars exhibited the highest migration speeds. Both platforms exhibited comparable speeds in the x and y directions, consistent with their trajectories. By contrast, cells on the grating with nanopillars displayed a reduced alignment angle compared to flat or nanopillar surfaces, reflecting more directional migration along the grating orientation. Compared to grating with nanopillars, the arrowheads with and without nanopillars exhibited similar alignment angles, indicating their migration directionality was about the same. The arrowheads with nanopillars demonstrated higher speeds in both the x and y directions, compared with the arrowheads without nanopillars. This suggests that the arrowhead pattern alone provides limited confinement of cells over the patterned area, and the nanopillar integration does not alter directional alignment in the absence of biochemical cues. Cells on FN coated the nanopillars of the arrowheads exhibited the smallest alignment angle, indicating the best directional guidance. Migration on this platform was strongly aligned along the arrowhead tips, indicating enhanced directionality guidance. Although grating with nanopillars exhibited the highest migration speed along the grating, they did not show the strongest directional alignment as observed for the FN coating platforms. This result is attributed to the biochemical confinement of cells over patterns introduced by the FN coating, which restricted lateral movement and promoted directional cell migration.

### 3.4. Arrowheads with Nanopillars and Fibronectin Coating Enhanced Directionality

**Figure 5:**
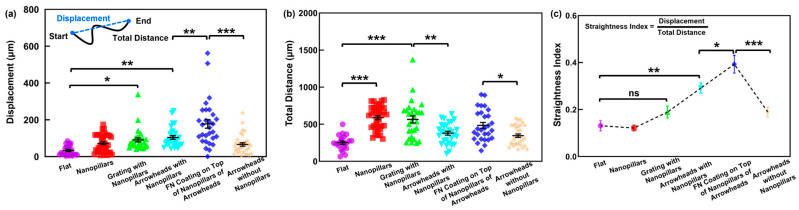
Analysis of cell migration directionality: (**a**) MC3T3 cell migration displacement; (**b**) cell total travelled distance; (**c**) cell straightness index on flat, nanopillar, grating with nanopillar, arrowheads with nanopillar, FN coating on top of nanopillars of arrowhead, and arrowheads without nanopillar surfaces. One-way ANOVA and Tukey’s post hoc test with * *p* < 0.05, ** *p* < 0.01, *** *p* < 0.001, and ns—not significant. Different colors correspond to groups of data on various platforms.

To further assess the directional guidance effect of the hPDMS platforms, both displacement and total distance were analyzed. Displacement was defined as the straight-line between a cell’s starting and ending positions, representing the distance travelled from beginning to end. Total distance represented the entire migration path, including all changes in direction, and served as an indicator of overall cell motility [22]. In Figure 5a, cells on flat surfaces showed a significantly lower displacement of 32.7 ± 5.9 μm due to the absence of directional guidance cues. Cells on nanopillars exhibited an increased displacement of 72.1 ± 8.0 μm due to the enhanced surface contact area for cell adhesion. The grating platforms with nanopillars showed a displacement of 90.8 ± 11.1 μm, slightly lower than that of the arrowheads with nanopillars. Cells on the grating platforms were able to extend along the grating ridges, resulting in longer migration paths. However, due to the symmetry of the grating, frequent 180° directional reversals occurred, leading to lower net displacement despite high motility [60]. For the arrowhead patterns, displacement increased from 65.8 ± 9.3 μm without nanopillars to 103.8 ± 10.0 μm with nanopillar integration, while total migration distance remained similar. This suggests that the addition of nanopillars enhanced guidance by increasing the contact area near the arrowhead tips, enabling more effective cell migration [21]. For the FN coated on nanopillars in the arrowhead arrangements, displacement further increased to 177.7 ± 23.5 μm. This large displacement was 1.9 times higher than that of the grating patterns and 2.7 times higher than that of the arrowheads without nanopillars. The combination of asymmetrical topography and biochemical coating was able to promote more directional cell migration along the arrowhead tips.

Previous studies have demonstrated that an FN coating on flat surfaces can increase adhesion and promote migration distance [61]. These effects were observed with the absence of topographical guidance cues. In the present study, grating patterns with 5 μm width and spacing provided continuous topographical cues that controlled the MC3T3 cell migration. However, coating the gratings with FN did not change their symmetrical geometry, and provided limited benefit in promoting unidirectional migration. By contrast, an asymmetrical topography with an FN coating introduces more FN interface near the arrowhead tips for cells migrating on the arrowhead patterns [62]. This design effectively reinforced persistent unidirectional migration, as evidenced by the substantial increase in the observed displacement.

In terms of the total migration distance, as shown in Figure 5b, cells on flat surfaces exhibited the lowest displacement of 307.3 ± 43.6 μm, which is expected given the absence of topographical or biochemical guidance cues. By contrast, cells on the nanopillar substrates and the gratings with nanopillars migrated significantly longer total distances of 587.9 ± 26.4 μm and 540.5 ± 42.3 μm, respectively. This substantial increase is likely attributed to the enhanced surface contact area provided by the dense nanopillars, which facilitated stronger cell interactions on platforms and greater motility. Cells on the arrowhead patterns with and without nanopillars showed comparable total migration distances of 379.2 ± 26.1 μm and 346.4 ± 23.2 μm, respectively. These results agreed with the previous migration speeds. Notably, the FN coating on the nanopillars with the arrowheads led to a significantly higher total migration distance of 484.7 ± 39.3 μm than the arrowheads without nanopillars, due to the FN confinement of cells on the patterns and the enhanced surface contact area by nanopillars that increased cell motility. These findings indicate that the total distance alone does not represent cell migration directionality.

To evaluate cell migration directionality, the straightness index was calculated as the ratio of displacement to total migration distance, as shown in Figure 5c. A value of 1 indicates that the cell migrated in a single direction without changes [5]. Flat and nanopillar surfaces showed a similar straightness index of 0.13 and 0.12, respectively, consistent with their isotropic features that resulted in cell migration in random directions. Although cells on the gratings with nanopillars had larger displacement than flat surfaces, their straightness index remained low due to bidirectional cell migration. This suggests that migration speed does not correlate with cell migration directionality. The arrowhead platforms coated with FN on top of the nanopillars exhibited the highest straightness index of 0.39, followed by the arrowheads with nanopillars of 0.29. By comparison, the arrowheads without nanopillars and gratings with nanopillars had a similar straightness index of 0.19. The FN coated platforms showed a 1.3 times increase in straightness compared to the uncoated arrowheads with nanopillars, and a 2.0-fold increase compared to the uncoated arrowheads without nanopillars. The straightness index results indicated that the asymmetrical patterned arrowheads induce the directional bias for cell migration by reducing movement in the opposite direction and guiding lamellipodia extension towards the tips of the arrowheads. Nanopillars further enhance this effect by increasing the effective surface contact area, which promotes the formation of more focal adhesions and enables traction forces to be transmitted towards the tips of the arrowhead patterns. The addition of an FN coating further strengthened this guiding effect by attracting cells on the patterned regions, thereby maintaining migration within the guidance track. Therefore, the asymmetrical topography using arrowheads provides the unidirectional cue, the nanopillars enhance the surface contact area, and the FN coating promotes interactions with the patterned surfaces, which collectively support unidirectional cell migration.

Moreover, even in the absence of the FN coating, the arrowheads with nanopillars provided a larger displacement and a higher straightness index than the gratings with nanopillars, suggesting that the arrowheads promote more directional cell migration. This effect could result from the asymmetrical distribution of focal adhesions and traction forces along the arrowhead axis, as opposed to the symmetrical distribution observed on the grating patterns. Previous research has indicated that FN alone could not induce directional bias on the symmetrical grating patterns, emphasizing the necessity of an asymmetrical topography to control cell migration direction [63]. Thus, the current findings demonstrate that the asymmetrical arrowheads enhance unidirectional cell migration, with the nanopillars and FN coating further boosting this effect by forming more focal adhesions around the arrowhead tips.

To further confirm the unidirectional guidance, cell migration distance along and against the arrowhead orientation were compared, as shown in Supplementary Figure S1. The migration distance on flat, nanopillar, and grating with nanopillar surfaces showed no significant differences along and against the pattern orientation, whereas the isotropic patterns and the symmetrical grating patterns provided equivalent guidance in both directions. By contrast, the arrowhead patterns induced significantly longer migration distances along the tip direction. This is attributed to the increased surface contact area near the tips of the arrowheads and the ability of cells to form larger protrusions in response to the asymmetrical cues [19,21]. The integration of nanopillars with arrowhead patterns further supported the formation of extended front regions along the arrowhead tips. The FN coating amplified this effect by chemically reinforcing directionality along the arrowhead tips. In this case, cells on the FN coated nanopillars of the arrowheads had the largest migration distance along the tip direction compared to other platforms, demonstrating the most unidirectional cell migration guidance. This observation is supported by the timelapse imaging in Supplementary Video SV1, which showed the MC3T3 cell migration on all the platforms. Cells on the platforms with FN coated on the nanopillars migrated with the best directional guidance along the tips of the arrowheads.

### 3.5. Focal Adhesion Distribution Influenced Unidirectional Cell Migration

To investigate how topographical cues influence unidirectional cell migration, cell morphology and FA distribution were analyzed. This study focuses primarily on topography-guided cell migration behavior, molecular mechanisms, such as signal transduction, gene regulation, and differentiation, remain to be explored. Future work will be expanded toward pathway-level investigations to complement the findings presented here. Focal adhesions serve as key regulators of traction sensing and transmission during cell migration [64]. They mediate mechanical force generation and are closely linked to directional movement [65,66]. Recent studies have demonstrated that the asymmetrical distribution of focal adhesions is strongly associated with persistent and directed cell migration [45,67,68]. To examine how mechanical forces contribute to unidirectional migration, the spatial distribution of FAs were analyzed in the front and back regions of the migrating cells. This analysis revealed the effects of the asymmetrical topography on cellular responses. Moreover, the FA distribution and area were analyzed and correlated with cell migration displacement for both symmetrical and asymmetrical topographies, showing their relevance to directional guidance mechanisms. Given that this study focused on understanding how asymmetrical cues promote unidirectional cell migration, the analysis emphasized conditions where directional cell migration could be observed.

Focal adhesion and F-actin staining were not performed on flat, nanopillar, or FN-coated flat surfaces, as previous studies have extensively characterized the effects of such platforms on cell adhesion and cytoskeletal organization [52,61,69]. It showed that, while these patterns enhance cell adhesion or spreading, they do not significantly affect directional migration behavior or promote persistent unidirectional movement [70]. Therefore, the immunofluorescence analysis in this study was focused on guiding patterns that provide directional cues. The immunostaining results from uncoated and FN-coated patterns are comparable. Focal adhesion and cytoskeletal analyses were conducted on three representative groups of the grating with nanopillars, the arrowheads with nanopillars, and the arrowheads without nanopillars. This approach allowed for a targeted evaluation of traction polarity, which is central to understanding unidirectional cell migration.

**Figure 6:**
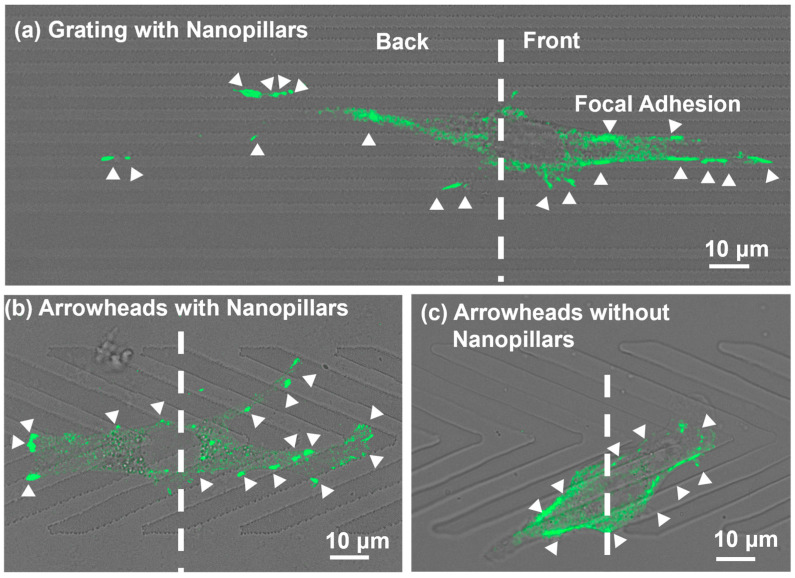
Immunofluorescence micrographs of focal adhesions (FAs) of MC3T3 cells migrating on (**a**) grating with nanopillars, (**b**) arrowheads with nanopillars, and (**c**) arrowheads without nanopillars. Green: vinculin. Cells were divided equally into front and back regions. White arrows indicate most of FAs on MC3T3 cells.

Figure 6 shows the representative vinculin fluorescence images of MC3T3 cells migrated on platforms featuring different topographical patterns. In Figure 6a, for the grating with nanopillars, cells exhibited elongated morphologies aligned with the grating ridge. Focal adhesions distributed evenly in the front and back regions, with similar numbers and sizes in both areas. This symmetrical distribution suggests that bidirectional traction forces are related to cell migration in both directions along the grating orientation. By contrast, for the arrowhead patterns, asymmetrical FA distributions were observed, as shown in Figure 6b,c. Focal adhesions were predominantly located in the front regions, especially on the arrowheads with nanopillars. This spatial bias corresponds to broader lamellipodia in the front regions and may result from the increased surface contact area near the arrowhead tips as provided by the arrowhead geometry. The asymmetry in FA localization suggests enhanced traction forces near the front regions, contributing to traction force imbalance and sustained unidirectional movement. Supplementary Figure S2 presents the measurements of cell area and aspect ratio. Cell areas remained comparable across all guiding platforms. However, cells on the grating with nanopillars appeared more elongated due to the dense and symmetrical pattern arrangement. The aspect ratio on the grating with nanopillars reached 6.3, compared to 4.5 and 4.9 on the arrowheads with and without nanopillars, respectively.

**Figure 7:**
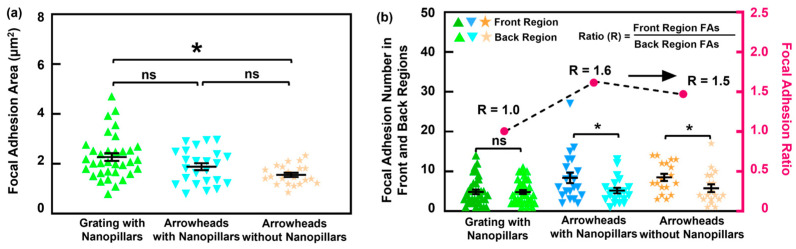
Analysis of FAs on MC3T3 cells migrating on grating with nanopillars, arrowheads with nanopillars, and arrowheads without nanopillars: (**a**) FA area and (**b**) number of FAs in front and back regions. One-way ANOVA and Tukey’s post hoc test with * *p* < 0.05 and ns—not significant. Different colors correspond to groups of data on various platforms.

A quantitative analysis of the FA area and distribution is shown in Figure 7. Cells on the grating with nanopillars had larger FA areas than those on the arrowhead patterns, as shown in Figure 7a. Focal adhesion areas were similar between the two arrowhead patterns with and without nanopillars. Despite this, the FA distributions were notably asymmetric on the arrowhead patterns, which agreed with the previous immunofluorescence images. Focal adhesions were concentrated toward the front regions, indicating that cells applied greater traction force in the direction of migration. The FA number ratio between the front and back regions on the arrowhead platforms was ≥1.5, with the highest ratio observed on the arrowheads with nanopillars. This suggests that the arrowheads with nanopillars increased the surface contact area at the front regions, promoting FA formation and enhancing forward motion along the tips of the arrowheads. By contrast, cells on the grating patterns displayed equal distribution of FAs in the two regions, indicating comparable traction forces at both ends of the cells. Such symmetry will cause the cells to move in both directions, despite alignment along the grating orientation. Supplementary Figure S3 further supports these observations. Even when FA numbers in the back regions were similar across platforms, the proportion of FAs in the front regions was significantly higher on the arrowhead patterns, especially those with nanopillars. This asymmetrical FA distribution likely contributes to traction force imbalance along the tips of the arrowheads, thereby supporting unidirectional cell migration.

### 3.6. Effects of Platform Topography on F-Actin Distribution

**Figure 8:**
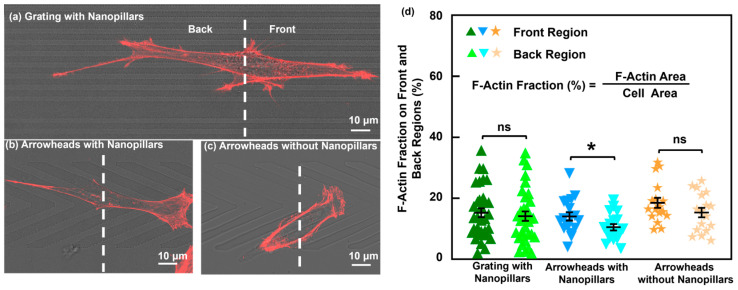
Immunofluorescence micrographs of F-actin of MC3T3 cells migrating on (**a**) grating with nanopillars, (**b**) arrowheads with nanopillars, and (**c**) arrowheads without nanopillars. Red: F-actin. (**d**) F-actin area fraction on front and back regions of cells. One-way ANOVA and Tukey’s post hoc test with * *p* < 0.05 and ns—not significant. Different colors correspond to groups of data on various platforms.

F-actin is a major structural component of the cell cytoskeleton and acts as a molecular force sensor, reflecting the mechanical interaction between cells and platforms [71,72,73]. As shown in Figure 8a, F-actin on the grating with nanopillars was symmetrically distributed between the front and back regions. This balanced distribution is consistent with comparable traction forces and supports bidirectional migration. However, cells on the arrowhead patterns exhibited polarized cytoskeletal organization, as shown in Figure 8b,c. F-actin was preferentially localized to the front regions. This asymmetry indicates cytoskeletal polarization, a feature of directed cell migration. A quantitative analysis of the F-actin distribution revealed similar fractions in both the front and back regions on the grating with nanopillars. This supports the observation of symmetric traction forces and cell migration in both directions. By contrast, the arrowhead patterns exhibited higher F-actin fractions at the front regions, especially more significant on the arrowheads with nanopillars. These results indicate that asymmetrical topographies, particularly when combined with nanostructures, induce cytoskeletal polarization and directional force generation. The preferential localization of F-actin in the front regions of the cell correlates with enhanced unidirectional migration, suggesting that topographical and nanoscale features directly influence cytoskeletal organization and the mechanical basis of cell guidance.

## 4. Discussions and Conclusions

In this study, the impact of micropattern, nanoscale topography, and biochemical modification on the cell migration direction of MC3T3 cells was systematically investigated. Micropatterned hPDMS platforms featuring symmetrical gratings and asymmetrical arrowhead patterns, with or without nanopillars or FN coating, were fabricated through NIL and hPDMS replication techniques. Both symmetrical gratings and asymmetrical arrowhead patterns were effective in guiding cell migration. However, asymmetrical arrowhead patterns were able to promote unidirectional cell migration due to their ability to induce cytoskeletal polarization and FA asymmetry in the front regions. Cells on arrowhead patterns demonstrated larger displacement and more directional migration compared to those on the gratings.

While previous studies have demonstrated the effects of asymmetrical patterns with an FN coating on cell migration in one direction, the current study aims to explore how nanotopography embedded in asymmetrical micropatterns and biochemical cues can work together to enhance unidirectional cell migration. The comparison between flat surfaces and nanopillars first confirmed that nanoscale features increase the available surface contact area for cells, leading to increased migration speed. Symmetrical gratings with nanopillars maintained bidirectional migration, highlighting the essential role of pattern asymmetry in establishing unidirectional movement. Introducing asymmetrical arrowhead micropatterns induced the necessary asymmetry, and combining them with nanopillars further increased the biased migration towards the arrowhead tip direction. Finally, an additional enhancement was observed with a selective FN coating on the tops of the nanopillars of the arrowheads, which was implemented to attract cells to the patterned regions. The results collectively support the conclusion that unidirectional migration was significantly enhanced through asymmetrical micropatterns, nanotopography, and selective biochemical coating. Although these multiple topographical guidance and FN coating approaches have been effective in controlling cell migration, additional studies will be needed to identify the interactions among these guiding elements.

The integration of nanopillars with the arrowhead micropattern significantly enhanced migration speed and overall cell displacement by increasing the surface contact area available for cell adhesion. A quantitative analysis showed that the nanopillars notably increased FA formation and enriched F-actin distribution in the cell front regions, further strengthening directional migration. Specifically, arrowhead patterns integrated with nanopillars exhibited a 1.6-fold larger displacement compared to the arrowheads without nanopillars, highlighting the positive effect of nanostructure integration to promote cell migration in a single direction. Moreover, the selective FN coating on top of the nanopillars of the arrowheads introduced biochemical confinement, substantially improving directional alignment and further enhancing cell displacement. The FN-coated nanopillars of the arrowheads demonstrated a 2.7-fold greater displacement and the highest straightness index compared to the unmodified arrowheads. This confirms that biochemical cues from the FN coating complement physical cues for the arrowhead platforms with integrated nanopillar topography, maximizing unidirectional cell migration.

A further consideration is the density of the grating and arrowhead patterns. The width, spacing, and symmetry of the patterns could influence cell migration behavior. The current findings indicated that topographical asymmetry is the dominant factor for directional cell migration. Both the grating and the arrowhead patterns have similar structures of 5 μm width and 5 μm spacing strips. In addition, the distance between the rows of arrowheads could potentially influence cell migration. Future studies could include symmetrical and asymmetrical patterns with various arrowhead row density to investigate the effects of row separation on cell migration directionality.

Another important consideration is the biochemical environment. While significant enhancement of unidirectional cell migration was achieved with the selective FN coating, only a single FN concentration was applied. Although this coated FN concentration exceeded the reported monolayer saturation threshold, plasma-treated platforms without the FN coating also had adsorbed proteins from the culture medium, introducing a background level of proteins. Future studies will be carried out to investigate how protein composition and concentration affect topography guided cell migration. These approaches will allow for a more precise evaluation of how protein concentration and spatial coverage could interact with topographical features to regulate directional cell migration.

FA and F-actin quantification were performed by dividing cells into front and back regions along their long axes using the Fit Ellipse function in ImageJ. The front region was assigned to the cell area with broader lamellipodia or larger cell membrane. For the grating patterns with nanopillars, cells migrated along the grating edges, and cell polarity and cell migration direction reversed frequently. As a result, similar FA numbers and F-actin fractions were observed in both the front and back regions. By contrast, the arrowhead patterns introduced asymmetrical geometry which biased cell polarization towards the arrowhead tips that resulted in more directional cell migration. The strongest unidirectional migration was observed on the platforms with FN-coated arrowheads with nanopillars. Moreover, the FA and F-actin analyses were conducted on uncoated PDMS platforms. These analyses provided information related to the density and distribution of FA and F-actin on various surface topographies. To study similar functionalization on FN coated platforms, different fluorescent markers that have an FN distinct emission wavelength from vinculin will be used. This would allow for the direct visualization of FA and F-actin on platforms coated with FN.

The platforms in this study were biomimetic and close to tissue structure for an in vitro analysis with precise control of the physical topographies and the biochemical coating. The grating and arrowhead patterns mimic symmetrical and asymmetrical structural cues, while the nanopillars resemble the nanoscale fibril structures in the ECMs. The selective FN coating further introduced biochemical molecules on the surface of the ECMs. This combination of controlled elements allows the platforms to capture the essential aspects of guidance cues while providing biomimetic platforms that can be precisely engineered. This combined strategy provides new insights into cell and material interactions and offers potential applications for engineering advanced biomaterial platforms that could promote targeted migration while minimizing nonspecific dispersion, such as enhancing the infiltration of immune cells to tumors or reducing scar formation.

## Data Availability

The original contributions presented in this study are included in the article/Supplementary Materials, further inquiries can be directed to the corresponding author.

## Supplementary Materials

The following supporting information can be downloaded at: https://www.mdpi.com/article/10.3390/jfb16090323/s1, Supplementary Figure S1: Analysis of cell migration distance of MC3T3 cells along and opposite to pattern orientations on PDMS platforms; Supplementary Figure S2: Analysis of cell area and aspect ratio of MC3T3 cells on the grating with nanopillars, arrowheads with and without nanopillars; Supplementary Figure S3: Analysis of FAs area in front region of MC3T3 cells migrating on the grating with nanopillars, arrowheads with and without nanopillars; Supplementary Video SV1: MC3T3 cells migrating on PDMS platforms.

## Abbreviations

The following abbreviations are used in this manuscript:

PDMS	Polydimethylsiloxane
FA	Focal adhesion
ECM	Extracellular matrix
FN	Fibronectin
IPS	Intermediate polymer stamp
FOTS	Trichloro(1H,1H,2H,2H-perfluorooctyl) silane
min	Minute
NIL	Nanoimprint lithography
UV	Ultraviolet
s	Second
rpm	Rotations per minute
RIE	Reactive ion etching
hPDMS	Hard polydimethylsiloxane
FBS	Fetal bovine serum
PBS	Phosphate saline buffer
h	Hour
PFA	Paraformaldehyde
DAPI	4′,6-diamidino-2-phenylindole
