# Supplementary material for: Enhanced Unidirectional Cell Migration Induced by Asymmetrical Micropatterns with Nanostructures
Source: J Funct Biomater. 2025 Sep 1;16(9):323. doi: 10.3390/jfb16090323 (PMC12470623; doi:10.3390/jfb16090323)
Supplement: Supplementary file 1 [file jfb-16-00323-s001.zip › jfb-3752522_Supplementary Materials.pdf]

Electronic Supplementary Information (ESI)

Enhanced Unidirectional Cell Migration Induced by Asymmetrical Micropatterns with Nanostructures

Kaixin Chen, Yuanhao Xu, and Stella W. Pang*
Department of Electrical Engineering
Centre for Biosystems, Neuroscience, and Nanotechnology
City University of Hong Kong, Hong Kong, China

*Corresponding Author:
S. W. Pang (pang@cityu.edu.hk)

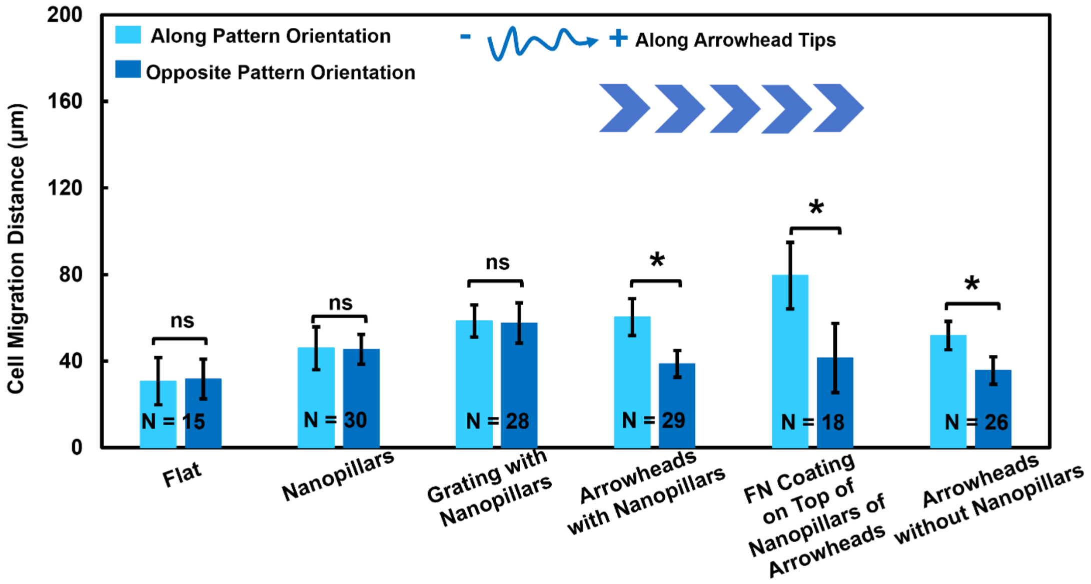

Supplementary Figure S1. Analysis of cell migration distance of MC3T3 cells along and opposite to pattern orientation on flat, nanopillars, grating with nanopillars, arrowheads with nanopillars, FN coating on top of nanopillars of arrowheads, and arrowheads without nanopillars. Migration distance represents total length of cell migration trajectory. One-way ANOVA and Tukey's post hoc test with *p <0.05 and ns – not significant.

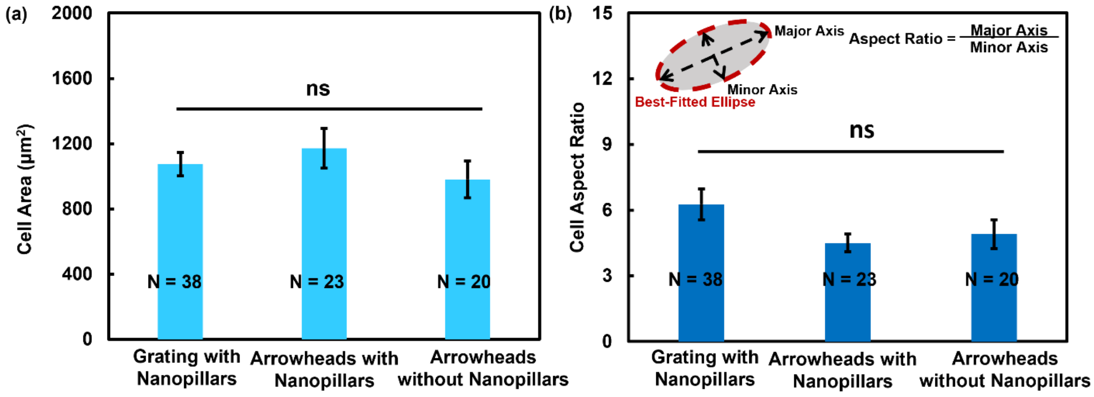

Supplementary Figure S2. Analysis of cell area and aspect ratio of MC3T3 cells on grating with nanopillars, arrowheads with and without nanopillars. Aspect ratio was calculated as ratio of cell's major axis to its minor axis. One-way ANOVA and Tukey's post hoc test with ns – not significant.

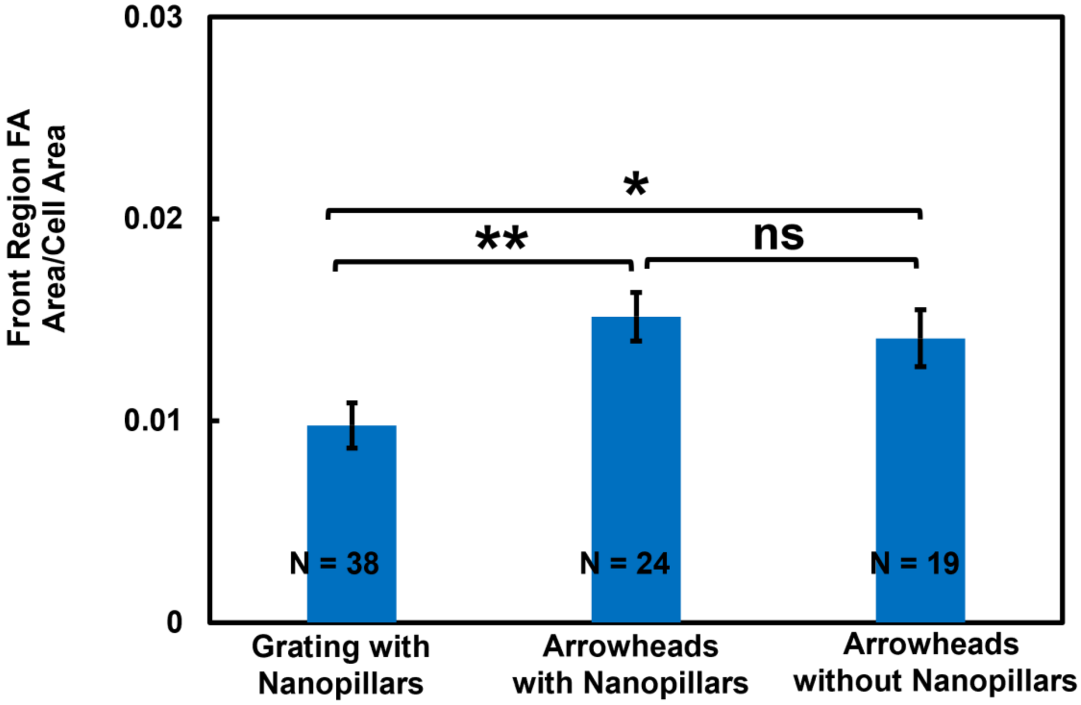

Supplementary Figure S3. Analysis of FAs area in front region of MC3T3 cells migrating on grating with nanopillars, arrowheads with and without nanopillars. One-way ANOVA with Tukey's post hoc test with *p <0.05, **p <0.01, and ns – not significant.

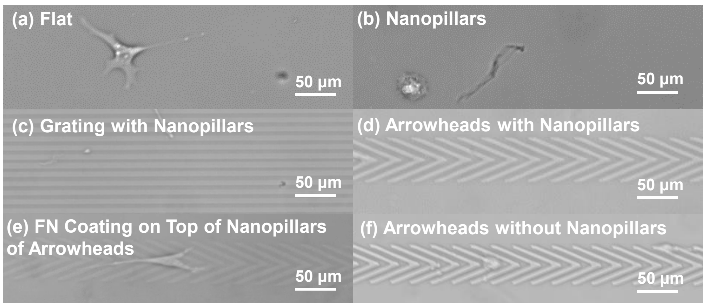

Supplementary Video SV1. MC3T3 cells migrating on (a) flat, (b) nanopillars, (c) grating with nanopillars, (d) arrowheads with nanopillars, (e) FN coating on top of nanopillars of arrowheads, and (f) arrowheads without nanopillars.
